# Putative bloodmeal sources in *Glossina austeni* tsetse fly of Arabuko Sokoke National Reserve in Kenya

**DOI:** 10.1371/journal.pone.0299243

**Published:** 2024-03-06

**Authors:** Kennedy O. Ogolla, Billiah K. Bwana, Clarence M. Mang’era, Tevin Onyango, Moses Y. Otiende, Benard Ochieng, Ahmed Hassanali, John M. Mugambi, Patrick Omondi, Paul O. Mireji

**Affiliations:** 1 Biotechnology Research Institute, Kenya Agricultural and, Livestock Research Organization, Kikuyu, Kenya; 2 Department of Biochemistry and Molecular Biology, Egerton University, Njoro, Kenya; 3 Wildlife Research and Training Institute, Naivasha, Kenya; National Veterinary Research Institute (NVRI), NIGERIA

## Abstract

Tsetse flies, the sole biological vectors of trypanosomiasis, are predominantly controlled using visual traps and targets baited with attractant lures. Formulation of the lures is informed by compositions of odors from vertebrate hosts preferred by specific tsetse species. However, there are no effective lures for *Glossina austeni*, a major vector of trypanosomiasis along eastern-coastal region of Africa. Formulation of the lure can be informed by knowledge of *G*. *austeni*, preferred vertebrate hosts. We thus sought to understand these hosts by assessment of putative bloodmeal sources of this tsetse fly in Arabuko Sokoke National Reserve where this species is naturally present. We sampled tsetse flies using NGU traps, isolated non-teneral *G*. *austeni* flies based on their feeding status, and identified vertebrate source of bloodmeals in their midgut contents using vertebrate 16S rRNA-PCR High-Resolution Melting analysis. We analyzed the relative vertebrate species frequencies in the bloodmeals using Fisher’s exact tests. Overall, we trapped 122 flies, most of which (66.39%) were non-teneral, among which we successfully identified the vertebrate bloodmeals in 30 samples. Specifically, we detected putative suni antelope (*Neotragus moschatus*), harnessed bushbuck (*Tragelaphus scriptus*), buffalo (*Syncerus caffer*) and cattle (*Bos taurus*) derived bloodmeals. Putative suni antelope bloodmeals were significantly more frequent (63.22%), than those of the harnessed bushbuck (23.33%), buffalo (10.00%) or cattle (3.33%) (*p* < 0.05 Fisher’s exact tests) among the samples analyzed. Suni antelope thus appears to predominate vertebrate bloodmeal source for *G*. *austeni* in the reserve, coincident with findings reported elsewhere, and is therefore a viable candidate for bioprospecting for *G*. *austeni* responsive attractants.

## Introduction

Tsetse flies are the sole biological vectors of trypanosome parasites, causative agents of Human African Trypanosomiasis (HAT) (sleeping sickness) in humans and African Animal Trypanosomiasis (AAT) (nagana) in their livestock, with dire health and economic implications in tsetse-infested regions of Africa [1, 2]. The HAT and AAT remains among most the Neglected Tropical Diseases (NTDs) in sub-Sahara Africa [3, 4]. In Africa, AAT is responsible for mortality of about three million cattle and loss of about US$ 4.75 billion per year in terms of agricultural Gross Domestic Product [5]. Trypanosomiasis has rendered livestock farming impossible in the most productive areas of Eastern Africa [6]. The disease impacts impoverished livestock farmers and threatens food security and livelihoods. There are no effective vaccines against the trypanosome parasites [7] and most field trypanosome isolates have developed resistance against available antitrypanosomal drugs [8, 9]. Prospects of using trypanotolerant cattle remain, but viability for wide scale adoption is not clear [10].

Among tsetse fly species, *Glossina austeni* and *G*. *pallidipes* are the major biological vectors of AAT along the East African coast [11]. Control of the flies constitutes the cornerstone of suppression and eradication efforts of sleeping sickness and nagana in sub-Sahara Africa [12]. Elimination of game animal hosts [13] and destruction of preferred habitats of tsetse fly [14] have been tried and abandoned due to their deleterious environmental effects. Application of synthetic insecticides appear to provide only a temporary solution to a permanent problem [15], manifested by tsetse re-infestation of cleared areas due to degradation of insecticide toxicity with time [13]. The sterile insect technique (SIT) is a powerful method of control typically in the final stages of Integrated Vector Management (IVM) for eradication of tsetse flies, first successfully applied in elimination of *G*. *austeni* in Zanzibar Island [16]. Success of SIT requires prior suppression of spatial density of tsetse flies using artificial baits, among other techniques [17], and is more effective when applied in islands where targeted tsetse population is isolated and the land is unexpansive. The baits consist of traps and targets that exploit visual and olfactory tsetse responses to odor cues from their hosts [18]. The host odors affect long distance attraction of tsetse flies to the baits, with visual stimuli required only for final orientation [19]. The success of this technology is therefore dependent on identification of appropriate visual and odors cues to which tsetse flies respond. All tsetse fly species are most attracted by blue, with black eliciting landing responses [20], phenotypic aspects of the fly that have informed the choice of color of the traps and targets. However, there are stark olfactory differences among tsetse fly species to odors emanating from their preferred hosts.

Consequently, odor from the fermented urine of buffalo (*Syncerus caffer*), a preferred host of *Glossina pallidipes*, *Glossina morsitans morsitans* and related savannah species of tsetse flies [21] was isolated and characterized to identify attractive constituents/compounds. This analysis revealed 3-propylphenol, octenol, p-cresol and acetone (POCA) blend as among the major tsetse attractive components of the odor compositions [22]. The POCA and more recently, a blend consisting of ε-nonalactone, nonanoic acid, 2-nonanone and acetone [23] have subsequently been adopted as olfactory bait for use in traps and targets for control of these flies. However, none of these blends is effective against *G*. *austeni*. Analysis of laboratory curated specimens suggest bushpig (*Potamochoerus larvatus*) [24] and field samples collected from the Shimba Hills National Reserve, Kwale, Kenya suggest suni antelopes as preferred hosts [25]. These different findings necessitated assessment of allopatric populations of the fly to validate either or both of these findings, or expand the range of possible preferred vertebrate hosts that can be bio-prospected for presence of potential *G*. *austeni* attractive odors.

We thus, undertook this study to assess vertebrate bloodmeal sources in allopatric populations of *G*. *austeni* in Arabuko Sokoke National Reserve, in the coastal region of Kenya.

## Materials and methods

### Ethical approval

We obtained approval to conduct this study from the Research Authorization Committees of the Wildlife Research and Training Institute (permit no: WRTI-0198-06-22) and Kenya Wildlife Service (permit no. KWS/BRM/5001).

### Study location

We conducted the study in the Arabuko Sokoke National Reserve (3°16′S 39°49′E), the largest coastal tropical forest in East Africa, covering about 420 square kilometers in Kilifi county, Kenya. The reserve is located 128 km from the Shimba Hills National Reserve and is populated with buffalo (*Syncerus caffer*), harnessed bushbuck (*Tragelaphus scriptus*), Aiders’s duiker (*Cephalophus adersi*), Sokoke bushy-tailed mongoose (*Bdeogale crassicauda*), savannah elephants (*Loxodonta africana*), African civets (*Civettictis civetta*), bush pigs (*Potamochoerus larvatus*), Sokokes (*Felis catus* and *F*. *silvestris lybica*), baboons (genus *Papio*), suni antelopes (*Neotragus moschatus*) and vervet monkeys (*Chlorocebus pygerythrus*), among other mammalian wildlife. The reserve is replete with a woodland (Brachyystegia), a forest (Cynnometra), and a composite of the two, each with different profiles of plants and wild animals. We conducted the study between November 24 and December 9, 2022, immediately after the wet season when tsetse flies are typically abundant.

### Study design, sample collection and putative vertebrate host species identification

**Fig 1:**
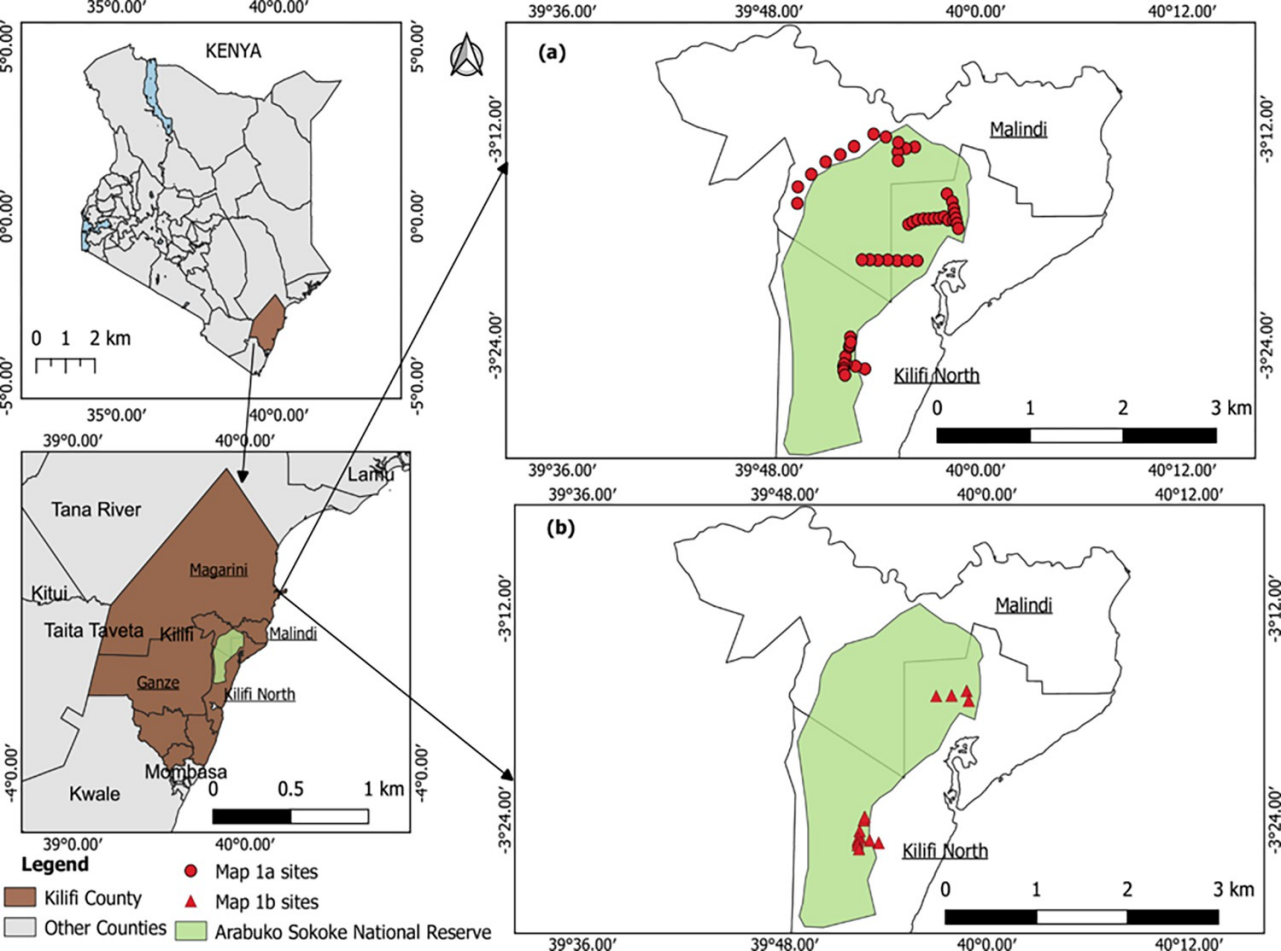
Map of Arabuko Sokoke National Reserve (green area), Kilifi County, Kenya, showing sampling areas for *G*. *austeni* tsetse flies. Map (**a**) shows the accessible sampled spatial locations/blocks within the reserve, while map (**b**) displays the locations/blocks where we trapped *G*. *austeni* tsetse flies. Map created using the Free and Open Source QGIS 3.22, made with Natural Earth.

Our study design, sample collection procedures and identification of source of bloodmeal were essentially as described by Ogolla et al. [25] with minor modifications. Briefly, we mapped and surveyed the reserve for assessment of relative abundance of *G*. *austeni* tsetse flies within human accessible sites. The tsetse flies typically occur in discreet pockets within their natural habitats and the reserve has tropical forests and thickets that limit access. About 60% of the reserve was accessible for sampling (**Fig 1A**), wherein we randomly placed NGU cloth traps [26] 1–5 km apart, collected flies trapped 24 hours post deployments and identified collected flies to species level using taxonomic keys [27]. This information (tsetse fly prevalence) informed our subsequent selection of blocks for definitive sampling (**Fig 1B**). In this definitive sampling, we deployed 60 NGU traps and 15 sticky panels [27] around tree trunks at intervals of at least 100 meters and collected the flies at 1700 hours each day for seven consecutive days. The trapping sites were spatially distributed in three of the blocks (**Fig 1B)**. We deliberately used unbaited sampling devices since there are no effective odor lures for *G*. *austeni*, thereby tsetse trapping fully relied on short-range visual (color) attraction to traps [28]. We then used taxonomic keys, based on size, color and male genitalia among other features [29], to confirm species of the *G*. *austeni* samples collected. The features included 1) projection of median lobes beyond general line of the superior claspers, 2) sandy reddish-brown dorsal surface adnominal colors, 3) lack of strong banding on abdomen, and 4) dark color on hind tarsi which extends to the last two tarsal segments. We established status of their feeding visually, assessed contents of their guts by dissection and microscopic examination, and preserved the samples in Whatman^®^ filter paper No. 1 as previously described [25]. Briefly, we cut out discs of about 1 cm diameter from blood spots on the filter papers, shredded them into finer fragments and separately placed them into 1.5 ml centrifuge tubes for DNA extraction from individual *G*. *austeni* samples. We then extracted genomic DNA using extraction kit (Bioline, London, UK) following the manufacturer’s instructions. We employed polymerase chain reaction (PCR) coupled with high resolution melting (HRM) analysis of the vertebrate 16S rRNA gene product as described by Ouso et al. [30] as amended by Ogolla et al. [25] for host bloodmeal identification, using a PCR-HRM thermal cycler (Qiagen, Germany). Based on the superior sensitivity of the 16S rRNA vertebrate mitochondrial gene in detecting host bloodmeals relative to cytochrome b gene as we previously established [25], we amplified the 16S rRNA gene with Vert 16S (Forward primer 5’-GAGAAGACCCTRTGGARCTT-3’ and Vert 16S Reverse primers 5’ -CGCTGTTATCCC TAGGGTA-3’), targeting approximately 200bp region. We used 15μl reaction volumes constituting of 4μl Hot firepol 5X evergreen (Master Mix), 0.5μl of 10 pmol vertebrate rRNA gene forward and reverse primers [31], 8μl of PCR grade water and 2μl of template DNA. We set the touch-down PCR amplification conditions as follows: initial holding temperature at 95°C for 15 minutes followed by 10 cycles at 94°C for 30 seconds, 63.5°C for 30 seconds and 72°C for 45 seconds. We set another 25 cycles at 94°C for 30 seconds, 50.5°C for 30 seconds and 72°C for 45 second, and the final extension at 72°C for 10 minutes. We included both positive (template) and negative controls (non-template) for assay quality assurance. We then conducted PCR and HRM analyses using Rotor-Gene Q software version 2.1.0.9. We conducted HRM ramping from 75°C to 95°C, rising by 0.1°C each step with a wait of 2 seconds for each step afterwards, as described by Nyamota et al. [32]. We analyzed HRM profiles using the Rotor gene 2.1.0.9 software, with normalized regions between 78°C and 92°C. We purified amplicons representative of each unique HRM profile using ExoSAP-IT™ (Applied Biosystems) as instructed by the manufacturer. We submitted 16 amplicons from individual *G*. *austeni* samples to Inqaba Biotec™, South Africa, for unidirectional Sanger sequencing.

We established the putative vertebrate hosts associated with the gene products by processing the sequences using Bioedit version 7.0.5.3 software [33] and searching the sequences against the National Center for Biotechnology Information (NCBI) non-redundant (nr) nucleotide database (https://www.ncbi.nlm.nih.gov/nucleotide/) (accessed on March 20, 2023) using the Basic Alignment Search Tool (BLAST) [34] where we accepted ≥99% homology among the sequences (subject and query) as identical.

### Data analysis

The focus of our analysis was to determine differences in feeding patterns of *G*. *austeni*, between the different vertebrate species identified. We based this on counts (frequencies of occurrences) of hosts identified in our sampling. We therefore employed Fisher’s exact test as some individual observations had less than five counts, to compare putative host associated with bloodmeals among the vertebrates. We considered *P* < 0.05 as significant in the Fisher’s exact tests analyses. We conducted all analyses using IBM SPSS version 22.0 (IBM Corp, Armonk, New York, USA).

## Results

**Fig 2:**
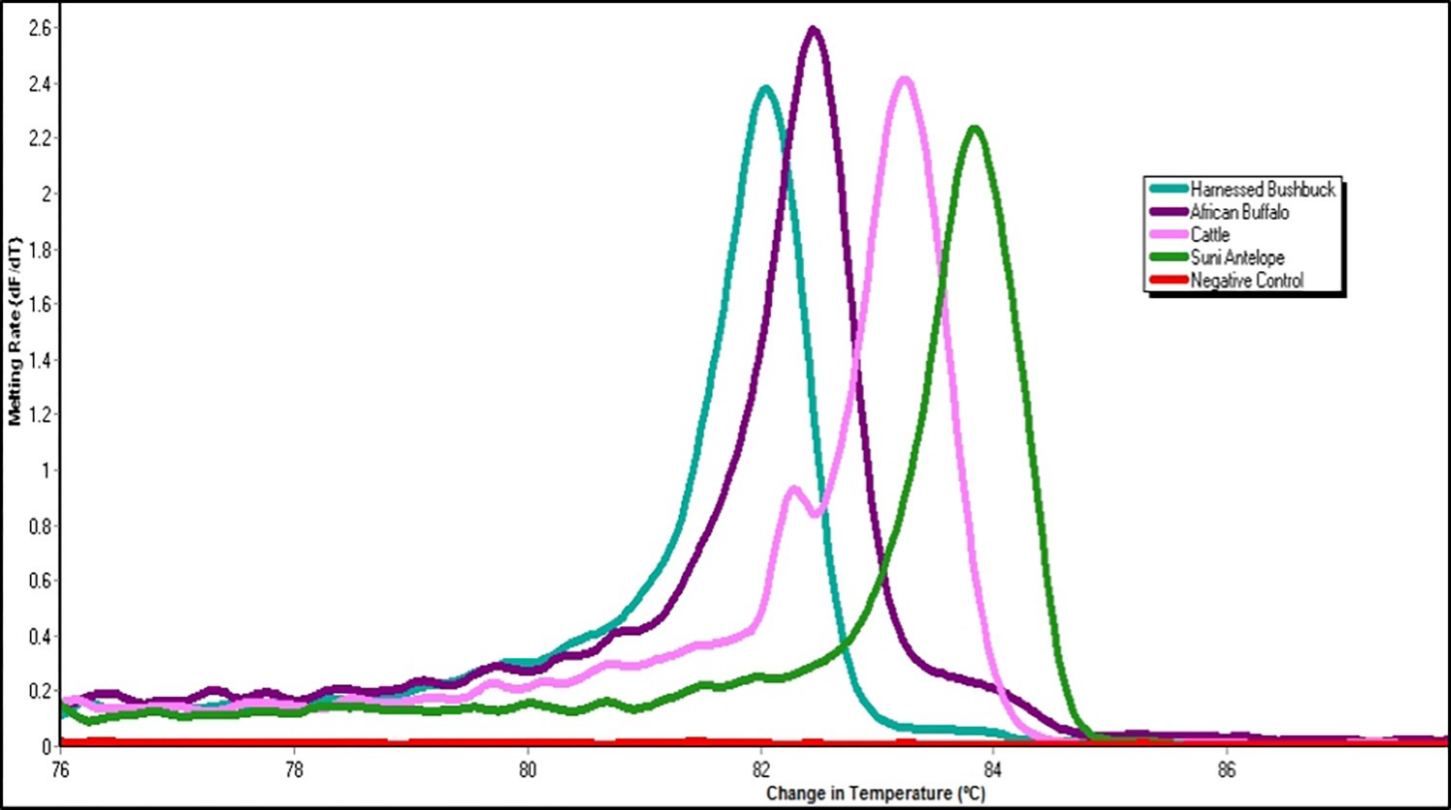
Distinct PCR-HRM profiles for putative vertebrate host species showing differentiation of bushbuck, antelope, buffalo, and cattle. The normalized HRM is for 16S rRNA markers represented as percent fluorescence. (NTC-Negative control).

We collected 122 *G*. *austeni* flies from the 60 traps, which included 97 females (34 tenerals and 63 non-tenerals) and 25 males (7 tenerals and 18 non-tenerals). In addition to tsetse flies, we trapped a total of 304 biting flies from the genera *Haematopota*, *Tabanus* spp., *Stomoxys* spp. and *Musca* spp. (**S1 Appendix**). Of the trapped flies, 81 (66.39%) were non-teneral, out of which 50 (61.73%) had bloodmeals (all trapped from two blocks, **Fig 1B**). We successfully identified vertebrate DNA in bloodmeals from 30 (60.0%) *G*. *austeni* tsetse flies. We detected putative suni antelope (*Neotragus moschatus*, 16S RNA GenBank accession JN632669), harnessed bushbuck (*Tragelaphus scriptus*, 16S RNA GenBank accession JN632707), African buffalo (*Syncerus caffer*, 16S RNA GenBank accession JQ235547) and cattle (*Bos Taurus*, 16S RNA GenBank accession OK183899) derived bloodmeals in the guts (**Fig 2**).

The majority (63.22%) of bloodmeals were derived from suni antelope, with those derived from harnessed bushbuck, African buffalo and cattle constituting 23.33, 10.00 and 3.33%, respectively. The proportion of bloodmeals from suni antelope was significantly higher (Fisher’s exact test) compared to harnessed bushbuck (*p* < 0.03), buffalo (*p* < 0.001) and cattle (*p* < 0.001). In all the bloodmeal samples, only a single putative vertebrate host species was detected.

## Discussion

In this wet season cross-sectional study, we established that the majority of the bloodmeals in *G*. *austeni* tsetse flies were putatively derived from suni antelope, with harnessed bushbuck as another major putative source of the bloodmeals for this species. There is presently no effective attractant formulated against this fly, consequently reducing efficacy of the existing artificial bait technology to control the fly. Previous effective attractants formulations such as POCA and novel attractant blend (ε-nonalactone, nonanoic acid, 2-nonanone and acetone) against savannah species have been informed by compositions of their preferred *Bovidae* hosts for blood meal analysis [23, 35]. While the trapped *G*. *austeni* sample size in the present study was small due to challenges with trapping the flies, the finding appears to resolve conflicting previous reports that presented suni antelope [25] or bushpig [24] as putatively preferred hosts of this fly, in support of the suni antelope. This is particularly important because the current findings and those of Ogolla et al. [25] represent allopatric populations of the fly, suggesting preferential selection of the antelope by the fly even in different ecological/geographical settings. The antelope should therefore be the most preferred candidate for isolation of *G*. *austeni* tsetse fly specific responsive odor, among the putative vertebrate hosts identified to date. However, the specific odor components of the antelope that are most attractive to the fly are yet to be identified and validated though laboratory, choice wind tunnel and field based behavioral studies. Previous studies on responses of savannah tsetse flies such as *G*. *pallidipes* and *G*. *m*. *morsitans* revealed that short and long-distance attractant molecules emanated from the breath, urine, skin and other parts of the vertebrate hosts [21, 22, 36].

Detection of cattle bloodmeals in *G*. *austeni* sheds light on the potential role of this fly species in local animal trypanosomiasis transmission. Since the current artificial bait technology is relatively inefficient in trapping this fly, transmission by the fly can persist undetected, particularly where the fly occurs in sympatry with other tsetse fly species such as *G*. *pallidipes* that are efficiently controlled by these devices. Formulation of an attractant effective against *G*. *austeni* and probably other sympatric tsetse fly species would significantly contribute to addressing this challenge.

In conclusion, the suni antelope appears to be most predominant vertebrate bloodmeal source for *G*. *austeni* among vertebrate hosts in the reserve. This finding supports a similar observation among allopatric populations of the same species in Shimba Hills National Reserve. The antelope is a likely viable candidate for bioprospecting for odor attractant(s) effective against adult *G*. *austeni*.

## Supporting information

S1 Appendix Distribution of tsetse trapped based on age (teneral and non teneral), sex, site of trapping, samples collected, those analyzed for bloodmeal detection and vertebrate hosts identified. (XLSX)
